# Validation of the abbreviated indicators of perceived residential environment quality and neighborhood attachment in China

**DOI:** 10.3389/fpubh.2022.925651

**Published:** 2022-08-02

**Authors:** Yanhui Mao, Xinyi Luo, Shuangyang Guo, Mei Xie, Jing Zhou, Rui Huang, Zhen Zhang

**Affiliations:** ^1^Institute of Applied Psychology, Psychological Research and Counseling Center, Southwest Jiaotong University, Chengdu, China; ^2^School of Foreign Languages, Southwest Jiaotong University, Chengdu, China; ^3^Dipartimento di Psicologia dei Processi di Sviluppo e Socializzazione, Sapienza Università di Roma, Rome, Italy; ^4^School of Economics and Management, Southwest Jiaotong University, Chengdu, China; ^5^School of Architecture and Design, Southwest Jiaotong University, Chengdu, China; ^6^Key Laboratory of Mental Health, Institute of Psychology, Chinese Academy of Sciences, Beijing, China

**Keywords:** Chinese urban community, neighborhood attachment, scale validation, measurement, perceived residential environmental quality

## Abstract

The purpose of this research is to utilize factor analyses to evaluate the reliability and factorial structure of an abbreviated version of the instrument that includes indicators of perceived residential environment quality (PREQ) and neighborhood attachment (NA) in Chinese urban environments. The instrument has 11 scales that measure PREQ and 1 scale measuring neighborhood attachment (NA). Architectural and urban planning aspects (three scales: *Architectural and Town-planning Space, Organization of Accessibility and Roads, Green Areas*), socio-relational aspects (one scale: *People and Social Relations*), functional aspects (four scales: *Welfare Services, Recreational Services, Commercial Services, and Transport Services*), and contextual aspects (three scales: *Pace of Life, Environmental Health*, and *Upkeep and Care*) are all covered by the 11 PREQ scales. A total of 1,332 people living in Chinese urban cities completed a self-report questionnaire that included these 12 scales. A calibration sample and a validation sample that were randomly split from the total sample verified the factorial structures of this instrument, and the abbreviated instrument had acceptable reliability and validity. The validated abbreviated version of the PREQ and NA instruments allowed for a more reliable and manageable tool that might lessen respondents' exhaustion of a large number of items, this also contributed to the policy-making for urban planning and practical architectural design.

## Introduction

Residential environmental quality is fundamental to people's lives and has represented a topic of great attention in Environmental Psychology and other environmental studies (1, 2). It can be investigated from either an “objective” or a “subjective” level (3, 4). The former level concerns physical “hard” measures (through technological tools or objectively quantifiable indicators) or expert evaluations based on specific professional backgrounds. While the second level concerns “soft” measures, which are dependent on individual perceptions and attitudes toward environmental quality. In the present study, we focus on the subjective level of residential environmental quality—perceived residential environment quality (5–7), and try to link it with one's perceived environmental correlate of neighborhood satisfaction—neighborhood attachment (8).

These two constructs, namely, perceived residential environment quality and neighborhood attachment, have been used to assess residential satisfaction—one of the most significant psychological patterns in Environmental Psychology (9, 10). Within the *Theory of Place*, residential satisfaction has been characterized as the sensation of fulfillment and pleasure of living in a particular area. It may capture the more considerable global experience of residents with their living environment (11). From an operational approach, residential satisfaction may be seen as a complex dimension (12), since it encompasses three fundamental components of the psychological construct of attitude: cognition, affection, and behaviors (13). This research investigates the cognitive and emotive components of residential satisfaction, which are assessed independently by two significant constructs: indicators of perceived residential environment quality and neighborhood attachment.

### Perceived residential environment quality

Perceived residential environment quality (PREQ) has received much attention as it is related to residents' perceived restoration, physical activity levels, civic behaviors, quality of life, and well-being (14–16). It stems from a more general study on perceived environmental quality indicators, concerned with people's perceptions of social-physical environmental characteristics (17). PREQ has been grasped by the analysis of residents' subjective assessment of the environmental features of their residential neighborhood. In contrast, the “subjective” and “objective” assessments of environmental characteristics may be uneven. Hence, it is beneficial to pay attention to the appraisal of environmental quality by people who reside in a specific location (4). People's evaluations of neighborhood quality, according to some scholars, encompass three primary components (18, 19): spatial (i.e., architectural and urban planning), human (i.e., people and social interactions), and functional (i.e., services and facilities). Later, environmental psychologists offered a fourth evaluative factor called contextual (pace of life, environmental health vs. pollution, and upkeep/care) (20), which was experimentally proven in many Italian cities (21, 22). It's worth noting that these four micro-evaluative dimensions of residential environment quality include almost all of the WHOQQL Group's environmental aspects of quality of life (23).

### Neighborhood attachment

Neighborhood attachment (NA) is considered as an affective component of residential satisfaction. It has been characterized as the positive feelings, relationships, ideas, and behavioral intentions that individuals establish through time with their social and physical surroundings, and it is referred to a narrowed concept under place attachment that indicates attachment to a specific geographical place (24). The residential neighborhood could be regarded as the most critical area in people's lives because of its importance in both temporal and relational terms. Therefore, neighborhood attachment, an essential indicator of community sustainability, affects social and residential well-being in different age groups (25, 26). From an operational viewpoint, neighborhood attachment has been operationalized as the propensity to offer a positive review of the residential area, the desire to improve it, and the reluctance to leave it (22). Although neighborhood attachment may comprise diverse elements via the different lenses, theoretically, it has been condensed to a one-dimensional concept by some scholars (9, 10).

### Association between PREQ and NA

Much research has been dedicated to perceived residential environmental quality and neighborhood attachment, as the former is associated with the latter (9, 10, 27). For example, people feel satisfied and attached to specific environments with good environmental qualities, such as the presence of social relationships, aesthetically pleasant buildings, and quiet and well-equipped green areas (9, 28). To this extent, perceived environmental quality can be considered the predictor of neighborhood attachment (9). However, in some other research, environmental quality can be predicted by place attachment, in that the residents who are attached to their neighborhood would indicate their residential area with good environmental quality (27), show pro-environmental behaviors such as waste recycling (29) and they are unwilling to move away from it even in coping in natural hazard contexts (30). Moreover, as stated by Bonaiuto et al. (9), the reciprocal relevance of the two constructs, namely, perceived residential environmental quality and residential attachment, should have specific psychological significance since it might act as a connection between two distinct levels of the psychological experience of individuals with their living locations. Residential environmental quality, in particular, has typically focused on issues of perception of specific components of urban environmental quality. In contrast, residential attachment has usually concentrated on molar aspects of the person-environment interaction.

From a methodological perspective, it is essential to have reliable and valid instruments measuring these two constructs (31). The instruments investigated in the study include an array of scales measuring perceived residential environmental quality (PREQ) and one scale measuring neighborhood attachment (NA). More specifically, PREQ indicators are standard indicators for evaluating a specific category of locations that have been used for academic purposes, policymakers, and practitioners (32), and they comprise 11 scales covering four above-mentioned macro-evaluative dimensions of residential quality (33). The first dimension, concerned with architectural and urban planning aspects of residential quality, is divided into three scales (*Architectural and Town-planning Space, Organization of Accessibility and Roads*, and *Green Areas*). The second dimension deals with interpersonal relationships and has just one scale (*People and Social Relations*). The third dimension relating to functional aspects is covered by four scales (*Welfare Services, Recreational Services, Commercial Services*, and *Transport Services*). The last dimension, which deals with the context, is made up of three scales (*Pace of Life, Environmental Health*, and *Upkeep and Care*). Table 1 presents some key contributing publications implicitly or explicitly covering the scales as mentioned above.

**Table 1:** Comparative study table with the major contributing publications.

**Source**	**Method**	**Results**
Bonaiuto et al. (9) (*N* = 497)	Using a multidimensional questionnaire for the measurement of PREQ and a unidimensional scale for NA; participants are inhabitants of 20 different neighborhoods in the city of Rome in Italy.	This model shows both the relevance of predictors from all four areas in predicting NA, and also a hierarchy between the areas in the power of the prediction (context area giving the most powerful predictors, services giving the weakest ones, architectural and town-planning, and social relations having intermediate importance). The instrument has 108 items in total.
Bonaiuto et al. (21) (*N* = 312)	These instruments consist of 11 scales measuring the PREQ of urban neighborhoods and one scale measuring NA; participants are residents in seven neighborhoods (differing in various features) of a great urban context like the city of Rome.	Results confirm the factorial structure of the scales, which include 19 perceived quality indices (150 items in total) and one NA index (8 items). The instrument has 158 items in total.
Bonaiuto et al. (22) (*N* = 1,488)	The instruments consist of 11 scales measuring the PREQ indicators and one scale measuring NA. The instruments consist of a self-report questionnaire, residents varied from different neighborhoods of 11 Italian middle- and low-extension cities (from 50,000 to 400,000 inhabitants).	Results confirm the factorial structure of the scales including 19 PREQ and 1 NA scales. A total of 148 items are included in this tool.
Fornara et al. (33) (*N* = 1,488)	Residents in various neighborhoods of 11 Italian middle- and low-population cities filled in a questionnaire including 12 scales (158 items), which corresponded to 11 PREQ and 1 NA scales.	Results showed good fit indices for factorial structures including overall 19 PREQ and 1 NA indicators, each one composed of three or four items, the abbreviated version consists of 66 items.
Sam et al. (34) (*N* = 466)	Inhabitants of 25 different neighborhoods in three different districts' municipal areas of the metropolitan municipality of Bursa (Turkey) completed the PREQ and NA indicators.	Neighborhood attachment was dominantly configured by the contextual, functional, and human features of the environment. Spatial features seemed to be less important. A total of 116 items are included in this tool.
Bonaiuto et al. (27) (*N* = 239)	The instruments consist of 11 scales measuring PREQI, one scale measuring NA, and three items about RS. PREQIs, NAS, and RS items are included in a self-report questionnaire (translated from English into Farsi language); participants are residents of Tabriz, Iran.	Multivariate statistical analyses of the survey results extend the cross-cultural validity of the tools, as well as testing relationship models going from specific to global PREQ Indicators, to NA Scale, finally predicting Residential Satisfaction. A total of 61 items are included in this tool.
Mao et al. (10) (*N* = 340)	The instruments consist of 11 scales measuring the PREQI and 1 scale measuring NA. The urban residents are from six districts (differing along with various features) of a highly urbanized context in Chongqing, China.	Results confirmed the factorial structure of the scales and demonstrated good internal consistency of the indicators, thus reaffirming the results of previous studies carried out in Western urban contexts. A total of 100 items are validated.
Debek and Janda-Debek (7) (*N* = 200)	Participants in Poland completed a commonly accepted and oft-cited questionnaire for measuring perceived urban environmental quality, the PREQ and NA Indicators.	The results of our study demonstrated a factorial validity of the tool's Polish language version relative to both the Italian original and its recent Iranian adaptation. A total of 42 items are included in this tool.
Ferreira et al. (35) (*N* = 110)	Participants in Sweden, completed a web-based survey, including measurements of walking intentions and behaviors, and the short version of both the PREQIs and NAS.	Structural Equation Modeling revealed direct effects of individual factors and neighborhood spatial-physical and social environmental qualities on transport walking. A total of 36 items are included in this tool.
Fornara et al. (36) (*N* = 383)	Participants in Paris filled in a questionnaire including the French version of the extended PREQIs and NAs scales.	PREQIs are validated in France with 139 items and 19 indicators (plus one indicator composed of 8 items for place attachment). The path analysis model presents an indirect connection between some PREQ and NA indicators via pace of life indicators, which are influenced by PREQIs and are directly associated with NA.
Zhang and Zhang (37) (*N* = 720)	The Chinese elderly participated in the study that investigated the relationship between perceived neighborhood environment and subjective well-being and the mediating effect of a sense of community.	Older adults participated in the study that investigated the relationship between perceived neighborhood environment and subjective well-being and the mediating effect of a sense of community. The instruments include a series of scales: SWLS (5 items), MIL (8 items), PANAS (20 items), Sense of Community Scale (10 items), and PRES (12 items).
Mao et al. (28) (*N* = 508)	Chinese residents were investigated in this study to explore how the spatial dimensions of PREQ, activity experience (i.e., flow) and social capital, would impact urbanities' residential community identity during COVID-19.	The result of structural equation modeling suggested that: a better degree in the spatial dimensions of PREQ would predict a stronger community identity; flow and social capital mediated the relationship between the spatial dimensions of PREQ and the residents' community identity.

### The present study

To this end, the instrument comprising both PREQ and NA indicators was used in previous studies carried out in different Italian urban areas, ranging from the large cities (9), to the small and medium-sized Italian cities (22). Subsequently, it was validated in different geographical and cultural areas within the European Union (e.g., France, Poland, Sweden) (7, 35, 36), then in far eastern cultural contexts (i.e., Turkey and Iran) (27, 34), and recently in China (10, 28, 37) which represented by far the most different and distant context from Italy (or other countries) in cultural, linguistic, and geographical terms. However, this instrument needs to be improved, as the number of the pertained items is large and the meaning of some items is similar or repetitive. Considering that the subjective evaluation of environmental quality is substantially related to people's well-being (38, 39), a more abbreviated instrument with shorter and more concise indicators would be more appealing to academics, practitioners, policymakers, and so on, which can reduce respondent fatigue because of the challenging usage in batteries of items. As a result, the current study attempted to verify an abridged form of this instrument that included the PREQ and NA indicators in a Chinese urban setting, based on the prior validation in China (10).

## Methods

### Sample and context

Study participants were 1,332 residents in the Chinese urban contexts who filled out our online survey via the spread survey link. They were 38.7% males and 61.3% females, and aged between 12 and 80 years (M = 30.36, SD = 11.69 years). We also got a large number of questionnaires from students, with 20.4% having a doctorate or master's degree, 61.9% having a bachelor's degree, 11% having a high school diploma, and 6.8% having just a middle school education. With regard to marital status, approximately 44.7% were single, 33.5% were married, 10.7% were in a romantic relationship, 9.5% were cohabitated, 1.4% were divorced, and 0.2% were widowed. Their self-reported socioeconomic income was 34.7% medium, 34.8% low, 23.2% medium-low, 5.7% medium-high, and 1.6% high. Residents who participated in the online survey were living in different Chinese urban cities, from the large cities, to the medium- and small-sized cities.

### Instruments

The instrument was included in a self-report questionnaire that had been translated from English to Chinese and then back-translated from Chinese to English by a professor of English language acquisition at Chongqing University, as described by Brislin (40). 11 PREQ Scales (93 items) and 1 NA Scale (7 items) were included in this self-report Chinese language questionnaire [see (10)]. Each scale contained both positive-worded items expressing environmental quality (e.g., “This neighborhood has good school facilities”) and negative-worded items expressing environmental quality (e.g., “The buildings have an unpleasant shape in this neighborhood”). The responses were graded on a seven-point Likert scale ranging from 1 (completely disagree) to 7 (completely agree). When constructing tests for cross-cultural and multilingual applications, measurement bias and sensitivity to confounding demographic and cultural variables have been carefully evaluated (41). As the most spoken language in the world, the Chinese language has its own contextual meanings, therefore, in order to fit this distinctive linguistic and cultural context, it's essential to make some modifications to the translated English version before administering the questionnaire.

The 11 PREQ and 1 NA Scales include the following 19 indicators of PREQ and 1 indicator of NA, as shown in Table 2.

**Table 2:** The original 100-item PREQ and NA scales (10) that need validation.

	**Scales**	**Factors**
PREQ	Architectural and town-planning space	1. Building volume
	(15 items)	2. Building aesthetics
		3. Building density
	Organization of accessibility and roads	1. External connections
	(10 items)	2. Internal practicability
	Green areas (6 items)	1. Green areas
	People and social relations (9 items)	1. Security and tolerance
		2. Sociability and cordiality
		3. Discretion and civility
	Welfare services (7 items)	1. Social care services
		2. School services
	Recreational Services (10 items)	1. Sport services
		2. Social-cultural activities
	Commercial services (6 items)	1. Commercial services
	Transport services (5 items)	1. Transport services
	Pace of life (11 items)	1. Relaxing versus distressing
		2. Stimulating versus boring
	Environmental health (7 items)	1. Environmental health
	Upkeep and care (7 items)	1. Upkeep and care
NA	Neighborhood attachment (7 items)	1. Neighborhood attachment

### Procedure

First, we put all translated items on a survey platform named *Questionnaire Star* to create an online questionnaire for a pilot test. After that, we sent the generated survey link and quick response (QR) code to the potential respondents by email and *WeChat* (the most widely and frequently used social media app in China) in order to obtain participation. Respondents who were invited to participate received written informed consent and completed the questionnaire with reference to their own neighborhood of residence. Ethical approval was obtained from Institutional Review Board at the first author's institute. The data collection phase ran from the mid of June to early November in the year 2021.

### Data analysis

Followed by the validation technique suggested by Fornara et al. (33), a cross-validation procedure [see (42, 43)] was pursued to create and validate the factorial structure of the PREQ and NA scales. We first of all, randomly split the sample (N = 1,332) into half, and the deviations between the two sets of data were verified by k-s test (44). We then used the first half of the sample (i.e., the calibration sample, *N* = 666) to construct a model via principal component analysis (PCA) with the software SPSS (26.0), and subsequently confirmed it on the second half (i.e., the validation sample, *N* = 666) by confirmatory factorial analysis (CFA) with the software Mplus (8.3).

Following the technique employed in the previous research (33), each scale was subjected to a step-by-step iterative analysis, beginning with the examination of an initial solution that included all of the items (each one loading just on the expected factor). A set of indices was used to assess the model's goodness of fit and, as a result, to determine whether to accept the solution that appeared at a given phase or to seek better solutions by lowering the number of items (45): the root-mean-square error of approximation (RMSEA) below the cut-off value of 0.08 (46), the standardized root-mean-square residual (SRMR) below the cut-off value of 0.08, the non-normed fit index (NNFI) and comparative fit index (CFI) above the cut-off value of 0.90, and finally a chi-square/degrees of freedom ratio of <3 (47).

The PREQ and NA indicators were calculated as the average of the observed values (i.e., the items). The relationship between NA and each of the PREQs was tested using Pearson's bivariate correlations. The internal consistency of the PREQ and NA was examined using Cronbach's alpha coefficient.

## Results

### Principal component analysis

**Figure 1:**
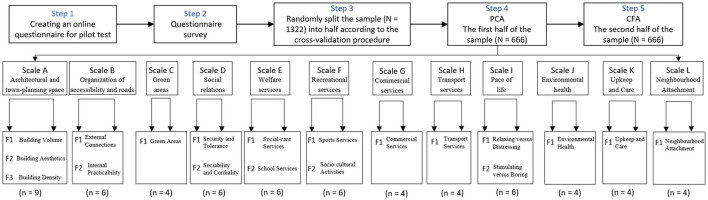
Research process and the PCA results (*n* is the number of items validated).

The factorial structure of the PREQ and NA scales was constructed using PCA on a random split-half sample of completed surveys (*N* = 666). To reduce the number of items per factor, a step-by-step iterative procedure was used for each scale, starting with analyzing an initial solution that included all the indicators/items (each one loading only on the expected factor). The number of indicators was set to three for multifactorial scales and four for mono-factorial scales, as suggested by Fornara et al. (33). The factor analysis results showed 18 PREQ factors with 59 items and 1 NA factor with 4 items. Figure 1 shows the findings of the factor analysis, as well as the research process.

### Confirmatory factor analysis

The factorial structure of the PREQ and NA scales was further validated using confirmatory factorial analysis in the second half of the sample (*N* = 666). Table 3 shows the CFA findings for each of the 11 PREQ scales covering the four macro evaluative dimensions of residential quality (architectural and urban planning, sociorelational, functional, and contextual factors) and one NA scale.

**Table 3:** CFA results for PREQ and NA scales (*N* = 666).

**Items**	**F1**	**F2**	**F3**
**Scale A: Architectural and Town-planning Space (*****n*** **=** **9)**			
13. Buildings are too large in this neighborhood	0.874		
14. The size of some buildings is excessive in this neighborhood	0.753		
12. Buildings are too tall in this neighborhood	0.712		
7. Buildings are unpleasant in this neighborhood		0.848	
6. Buildings have unpleasant colors in this neighborhood		0.828	
8. The buildings have an unpleasant shape in this neighborhood		0.743	
4. Buildings are too clustered in this neighborhood			0.863
1. Buildings are too close together in this neighborhood			0.829
2. There's little space between buildings in this neighborhood			0.802
Alpha	0.833	0.870	0.868
Fit indices: χ^2^ = 93.182; df = 24; χ^2^/df = 3.883; RMSEA = 0.066; SRMR = 0.035; NNFI = 0.966; CFI = 0.977			
**Scale B: Organization of Accessibility and Roads (n** **=** **6)**			
This neighborhood is well connected with important parts of the city	0.904		
The city center can be easily reached from this neighborhood	0.684		
24. There's a large choice of roads to get out of the neighborhood	0.492		
19. Parking places and parking lots are lacking in this neighborhood		0.830	
16. Parked cars impede walking in this neighborhood		0.649	
17. There is good availability of parking spaces in this neighborhood		−0.615	
Alpha	0.719	0.737	
Fit indices: χ^2^ = 22.109; df = 8; χ^2^/df = 2.764; RMSEA = 0.051; SRMR = 0.038; NNFI = 0.972; CFI = 0.985			
**Scale C: Green Areas (*****n*** **=** **4)**			
27. There are enough green areas in this neighborhood	0.898		
28. In this neighborhood green areas are in good condition 26. There are green areas for relaxing in this neighborhood 29. There is at least a garden/park where people can meet in this neighborhood	0.859	0.826	0.710
Alpha	0.876		
Fit indices: χ^2^ = 5.859; df = 2; χ^2^/df = 2.930; RMSEA = 0.054; SRMR = 0.008; NNFI = 0.993; CFI = 0.998			
**Scale D: Social Relations (*****n*** **=** **6)**			
32. Disreputable persons hang around in this neighborhood.	0.824		
34. Late in the evening there is the risk of dangerous encounters in this neighborhood.	0.814		
33. People often behave uncivilly in this neighborhood.	0.693		
39. In this neighborhood people tend to be isolated.		0.881	
38. In this neighborhood it is difficult to make friends with people.		0.838	
40. In this neighborhood people only have formal relationships.		0.779	
Alpha	0.740	0.870	
Fit indices: χ^2^ = 12.130; df = 8; χ^2^/df = 1.516; RMSEA = 0.028; SRMR = 0.016; NNFI = 0.996; CFI = 0.998
**Scale E: Welfare Services (*****n*** **=** **6)**			
45. Elderly care services are lacking in this neighborhood	0.849		
44. Social services are inadequate in this neighborhood	0.731		
46. The local health service is inadequate in this neighborhood	0.692		
43. Schools are generally good in this neighborhood.		0.792	
42. Schools can be easily reached on foot in this neighborhood		0.606	
41. This neighborhood has good school facilities		0.434	
Alpha	0.842	0.818	
Fit indices: χ^2^ = 20.482; df = 7; χ^2^/df = 2.926; RMSEA = 0.054; SRMR = 0.017; NNFI = 0.978; CFI = 0.990			
**Scale F: Recreational Services (*****n*** **=** **6)**			
48. You can do various sports in this neighborhood	0.823		
50. There are areas where you can do outdoor sports in this neighborhood	0.770		
51. If you like jogging, this neighborhood is suitable	0.759		
56. This neighborhood is well served to host theater performances		0.873	
The neighborhood is often animated by several cultural events (exhibitions, shows, etc.)		0.783	
57. In this neighborhood libraries are adequate for residents' needs		0.584	
Alpha	0.846	0.834	
Fit indices: χ^2^ = 30.731; df = 8; χ^2^/df = 3.841; RMSEA = 0.065; SRMR = 0.023; NNFI = 0.975; CFI = 0.987			
**Scale G: Commercial Services (n** **=** **4)**			
59. Anything can be found in the neighborhood's stores	0.880		
58. There are all kinds of stores in this neighborhood	0.717		
60. This neighborhood is well-served with stores	0.593		
63. In this neighborhood stores selling the most needed products can be easily reached	0.450		
Alpha	0.823		
Fit indices: χ^2^ = 7.205; df = 2; χ^2^/df = 3.603; RMSEA = 0.063; SRMR = 0.017; NNFI = 0.977; CFI = 0.992			
**Scale H: Transport Services (*****n*** **=** **4)**			
66. Buses are too uncomfortable in this neighborhood	−0.828		
67. The quality of public transportation is poor in this neighborhood	−0.811		
65. In this neighborhood the frequency of public transport is adequate for residents' needs	0.564		
68. The time spent waiting for public transport is too long in this neighborhood	−0.522		
Alpha	0.806		
Fit indices: χ^2^ = 0.810; df = 2; χ^2^/df = 0.405; RMSEA = 0.000; SRMR = 0.006; NNFI = 1.005; CFI = 1.00			
**Scale I: Pace of Life (*****n*** **=** **6)**			
69. There is a calm atmosphere in this neighborhood	0.874		
70. If compared with the chaos of other areas, this neighborhood is still liveable	0.639		
72. There is a peaceful pace of life in this neighborhood	0.516		
77. This neighborhood is very boring		0.835	
76. Nothing happens in this neighborhood		0.675	
78. Only a few things can be done in this neighborhood.		0.580	
Alpha	0.804	0.738	
Fit indices: χ^2^ = 13.706, df = 7; χ^2^/df = 1.958; RMSEA = 0.038; SRMR = 0.019; NNFI = 0.984; CFI = 0.993			
Scale J: Environmental Health (*n* = 4)			
83. Residents' health is threatened by pollution in this neighborhood	−0.833		
84. This is a polluted neighborhood	−0.772		
85. There is too much noise in this neighborhood	−0.712		
80. The air is clean in this neighborhood	0.433		
Alpha	0.817		
Fit indices: χ^2^ = 5.182; df = 2; χ^2^/df = 2.591; RMSEA = 0.049; SRMR = 0.012; NNFI = 0.988; CFI = 0.996			
**Scale K: Upkeep and Care (*****n*** **=** **4)**			
88. Road signs are well-kept in this neighborhood	0.758		
87. Streets are regularly cleaned in this neighborhood	0.720		
93. The refuse collection service is efficient in this neighborhood	0.584		
89. Residents show care for their neighborhood	0.528		
Alpha	0.786		
Fit indices: χ^2^ = 5.626; df = 2; χ^2^/df = 2.813; RMSEA = 0.052; SRMR = 0.015; NNFI = 0.981; CFI = 0.994			
**Scale L: Neighborhood Attachment (*****n*** **=** **4)**			
99. I have nothing in common with this neighborhood	0.842		
98. I would willingly live in another neighborhood	0.784		
97. I do not feel integrated into this neighborhood	0.696		
100. I do not subscribe to this neighborhood's lifestyle	0.596		
Alpha	0.835		
Fit indices: χ^2^ = 0.094; df = 2; χ^2^/df = 0.047; RMSEA = 0.000; SRMR = 0.001; NNFI = 1.006; CFI = 1.00			

Notes. n, the number of items; CFI, comparative fit index; NA, neighborhood attachment; NNFI, nonnormed fit index; PREQIs, Perceived Residential Environment Quality Indicators; RMSEA, root-mean-square error of approximation; SRMR, standardized root-mean-square residual.

#### Aspects of architecture and urban planning

The model included three linked factors for the scale measuring *Architectural and Town-planning Space* (see Scale A in Table 3). Building Volume (F1 in the table) was the first component, and it featured three items (all negatively phrased) relating to excessive building size. Building Aesthetics (F2) was the second element, and it included three items (all negatively phrased) about neighborhood pleasantness, building attractiveness, and building colors. Building Density (F3) was the third factor. It included three items (all negatively phrased) about the lack of enough space between buildings and the imbalance between built-up regions and open spaces. After removing six items from the original scale (two items respectively from each factor), the model fit indices were satisfactory.

The model contained two linked factors for the scale assessing *Organization of Accessibility and Roads* (see Scale B in Table 3). External Connections (F1 in the table) was the first factor, and it featured three items (all favorably phrased) about neighborhood connections to the city center and other city districts. Internal Practicability (F2) was the second component, and it included three items (one good and two negatives) about the ease of walking and cycling and parking availability. The original scale eliminated four elements (two items from each factor). This model's fit indices revealed a decent overall fit.

The model for the *Green Areas* scale (see Scale C in Table 3) was monofactorial, with four items (all favorably phrased) representing the availability and amount of green areas, as well as the opportunity of resting in them. After removing two items from this scale, the model fit was satisfactory.

#### Aspects of socio-relationships

The model included two associated factors for the scale evaluating *Social Relational Features* (see Scale D in Table 3). The first factor, Security and Tolerance (F1 in the table), included three items (all negative) related to the possibility of hazardous night gatherings, incivility, and dangerous persons. The second factor, Sociability and Cordiality (F2), included three negative items about a proclivity for formal interpersonal connections, isolation, low sociability, and bad friendship. When three items from the original scale were deleted, the results suggested that the model fit was satisfactory.

#### Aspects of functionality

The model comprised six items loading on two associated components for the scale assessing *Welfare Services* (see Scale E in Table 3). The first factor, Social Care Services (F1), had three negative items pertaining to the insufficiency of social, health, and aged care services. The second factor, School Services (F2), consisted of three items (all positive) that referred to the number and quality of schools in the neighborhood. To get acceptable fit indices, one item was deleted from the first factor.

The model comprised six items loading on two associated components for the scale evaluating *Recreational Services* (see Scale F in Table 3). The first factor, Sports Services (F1), included three items (all positive) on the availability of outdoor and indoor sports facilities in the community. The second factor, Sociocultural Activities (F2), had three items (all positive) on the existence of entertainment and cultural attractions. This scale reduced four items, with three things deleted from the first factor and one item removed from the second. The model showed an excellent fit.

The model contained four items (all positive) loading on the single factor of Commercial Services for the scale measuring *Commercial Services* (see Scale G in Table 3; F1). The items were related to the number, variety, and dispersion of local stores, as well as the ease with which they could be reached. To get an acceptable fit, two items were removed.

The model contained four items (one positive and three negative) loading on the single factor of Transport Services for the scale measuring *Transport Services* (see Scale H in Table 3; F1). The items discussed the public transportation system, as well as its regularity, variety, and comfort. To get a great fit, one item was deleted from the original scale.

#### Aspects of context

The model contained two associated factors for the scale evaluating *Pace of Life* (see Scale I in Table 3). The first factor, Relaxing vs. Distressing (F1), comprised of three items (all positive) that were linked to the neighborhood's tranquil and serene pace of life. The second factor, Stimulating vs. Boring (F2), had three negative items on the lack of fascinating and exciting neighborhood events and activities. To get a satisfactory overall fit, one item was deleted from the original scale.

The model contained four items (one positive and three negative) loading on the single factor of Environmental Health for the scale assessing *Environmental Health* (see Scale J in Table 3; F1). The items were related to air quality, pollution, and noise. After three items were eliminated from the initial scale, the model fit indices indicated a very excellent fit.

The model contained four items (all positive) loading on the single component of Upkeep and Care for the scale assessing *Upkeep and Care* (see Scale K in Table 3; F1). The items included both public and resident care for their neighborhood's roadways, road signs, and other amenities. To get a suitable fit, three items were deleted from the original scale.

#### Neighborhood attachment

The model comprised four items (all negative) loading on the sole factor of Neighborhood Attachment for the scale assessing NA (see Scale L in Table 3; F1). The items discussed the integration and identity of the resident's neighborhood. The original scale had three items deleted. This model's fit indices indicated a decent overall fit.

The internal consistency (Cronbach's alpha values) for each of the PREQ and NA scales is shown in Table 4.

**Table 4:** Summary of the abbreviated version of the Chinese PREQ and NA scales (*N* = 1,332).

**Generative criteria**	**Scales**	**Factors**	**No. of items**	**Alpha**
Architectural/town-planning features	Architectural and town-planning spaces	F1. Building volume	3	0.833
		F2. Building aesthetics	3	0.870
		F3. Building density	3	0.868
	Organization of Accessibility and roads	F1. External connections	3	0.719
		F2. Internal practicability	3	0.737
	Green areas	F1. Green areas	4	0.876
Sociorelational features	People and social relations	F1. Security and tolerance	3	0.740
		F2. Sociability and cordiality	3	0.870
Functional features	Welfare services	F1. Social-care services	3	0.842
		F2. School services	3	0.818
	Recreational services	F1. Sports services	3	0.846
		F2. Socio-cultural activities	3	0.834
	Commercial services	F1. Commercial services	4	0.823
	Transport services	F1. Transport services	4	0.806
Contextual features	Pace of life	F1. Relaxing vs. distressing	3	0.804
		F2. Stimulating vs. boring	3	0.738
	Environmental health	F1. Environmental health	4	0.817
	Upkeep and care	F1. Upkeep and care	4	0.786
Neighborhood attachment	Neighborhood attachment	F1. Neighborhood attachment	4	0.835

### Correlations between indicators of PREQ and NA

The statistical characteristics of the 18 PREQ indicators and one NA indicator are shown in Table 5. We utilized the whole sample (*N* = 1,332) in this stage. PREQ are sorted by the magnitude of their bivariate correlation with NA, with the greatest Pearson's correlations at the top and the lowest Pearson's correlations at the bottom. All of the relationships between NA and PREQ were statistically significant. All of the sociorelational PREQs had a substantial and high bivariate association with NA, including Sociability and Cordiality (*r* = 0.500, *p* < 0.001) and Security and Tolerance (*r* = 0.410, *p* < 0.001). Environmental Health (*r* = 0.508, *p* < 0.001), Stimulating vs Boring (*r* = 0.496, *p* < 0.001), Relaxing versus Distressing (*r* = 0.346, *p* < 0.001), and Upkeep and Care (*r* = 0.257, *p* < 0.001) were the four contextual characteristics of PREQ that exhibited substantial and strong associations with NA (from highest to lowest). Building Aesthetics (*r* = 0.417, *p* < 0.001), Building Density (*r* = 0.351, *p* < 0.001), Green Areas (*r* = 0.344, *p* < 0.001), Internal Practicability (*r* = 0.246, *p* < 0.001), Building Volume (*r* = 0.243, *p* < 0.001), and External Connections (*r* = 0.169, *p* < 0.001) all had significant and fairly high correlations with NA. The functional aspect of PREQ revealed one low-correlation indicator (Sociocultural Activities, *r* = 0.060, *p* < 0.05) and five indicators with a significant and moderately strong correlation with NA (*r* = 0.338, *p* < 0.001 for Social Care Services; *r* = 0.295, *p* < 0.001 for Transportation Services; *r* = 0.272, *p* < 0.001 for Sports Services; *r* = 0.198, *p* < 0.001 for School Services; and *r* = 0.138, *p* < 0.001 for Commercial Services).

**Table 5:** PREQ and NA indicators: mean, standard deviation, and correlational matrix (*N* = 1,332).

	**Variable**	**Mean (SD)**	**Correlation** **(NA-PREQ)**															
NA	1. NA	4.226 (1.028)	**1**	**2**	**3**	**4**	**5**	**6**	**7**	**8**	**9**	**10**	**11**	**12**	**13**	**14**	**15**	**16**	**17**	**18**	**19**
PREQIs	2. Environmental Health	4.541 (1.062)	0.508	**1**																	
	3. Sociability and Cordiality	4.379 (1.151)	0.500	0.455	**1**																
	4. Stimulating vs. Boring	4.109 (0.963)	0.496	0.424	0.446	**1**															
	5. Building Aesthetics	4.401 (1.112)	0.417	0.419	0.316	0.329	**1**														
	6. Security and Tolerance	4.620 (0.997)	0.410	0.544	0.466	0.300	0.399	**1**													
	7. Building Density	4.328 (1.258)	0.351	0.448	0.338	0.286	0.563	0.421	**1**												
	8. Relaxing vs. Distressing	4.587 (1.015)	0.346	0.578	0.266	0.220	0.302	0.382	0.3	**1**											
	9. Green Areas	4.357 (1.227)	0.344	0.478	0.274	0.294	0.401	0.387	0.395	0.456	**1**										
	10. Social Care Services	3.858 (1.187)	0.338	0.421	0.358	0.347	0.363	0.358	0.391	0.274	0.426	**1**									
	11. Transport Services	4.277 (1.058)	0.295	0.427	0.309	0.379	0.290	0.377	0.271	0.301	0.317	0.447	**1**								
	12. Sport Services	4.105 (1.262)	0.272	0.372	0.245	0.283	0.296	0.267	0.331	0.418	0.646	0.423	0.289	**1**							
	13. Upkeep and Care	4.502 (0.962)	0.257	0.493	0.238	0.232	0.317	0.355	0.338	0.563	0.539	0.411	0.411	0.507	**1**						
	14. Internal Practicability	3.942 (1.240)	0.246	0.361	0.215	0.201	0.462	0.349	0.457	0.265	0.459	0.405	0.255	0.395	0.34	**1**					
	15. Building Volume	4.291 (1.066)	0.243	0.279	0.275	0.176	0.370	0.251	0.421	0.202	0.099	0.229	0.21	0.099	0.124	0.183	**1**				
	16. School Services	4.380 (1.191)	0.198	0.322	0.193	0.183	0.225	0.257	0.24	0.359	0.42	0.324	0.335	0.452	0.438	0.268	0.131	**1**			
	17. External Connections	4.460 (1.049)	0.169	0.308	0.227	0.214	0.208	0.261	0.188	0.311	0.336	0.312	0.522	0.281	0.389	0.192	0.111	0.379	**1**		
	18. Commercial Services	4.070 (1.178)	0.138	0.174	0.132	0.190	0.190	0.097	0.169	0.288	0.406	0.354	0.343	0.47	0.436	0.271	0.012	0.414	0.365	**1**	
	19. Socio-cultural Activities	3.450 (1.301)	0.060	0.075	0.057	0.164	0.116	0.047	0.171	0.161	0.384	0.351	0.137	0.541	0.321	0.282	−0.078	0.339	0.192	0.502	**1**

Notes. NA = neighborhood attachment; PREQIs = Perceived Residential Environment Quality Indicators.

## Discussion

PREQ indicators (9) are a set of standard indicators that measure inhabitants' perceptions of the quality of their urban living environment (i.e., the urban neighborhood). NA is defined as the tendency to give a positive evaluation of the residential neighborhood, the motivation to improve it, and the reluctance to leave it (22). In many Italian regions, both PREQIs and NA indicators have been verified (9, 22), and subsequently in different cultural areas within the European Union (such as in France), then in far eastern cultural contexts (i.e., Iran). However, this instrument needs to be improved, as the number of the pertained items is large and the meaning of some items is similar or repetitive, especially it may encounter challenges in Chinese culture. Therefore, based on prior efforts (10), the present work validated a shortened version of the PREQ instrument for Chinese academics, practitioners, and policymakers. The validated shortened version eliminated some items that were repetitive and confusing. It yielded an abbreviated tool with more concise indicators that would be more suitable, manageable, and easy to use in Chinese urban contexts.

Compared with the prior validation carried out in Chongqing, China (10), participants involved in the present work were recruited in different neighborhoods of Chinese urban contexts, which could provide more generalized conclusions with the larger sample size. It is worth noting that a cross-validation procedure was conducted in the present work; that is, the sample was split into two half random samples, one for constructing the model based on the PCA technique and one for confirming the model with the CFA data analytical strategy. Compared with previous validation methods that used PCA or CFA separately (10, 21), this methodological choice could help select items more conservatively. In addition, the confirmatory nature of the present study throughout a cross-validation process was a further step in validating instruments measuring perceived residential environmental quality and neighborhood attachment.

The factor structures of the PREQ and NA indicators were concordant with the previous works (10, 28, 33), except for the People and Social Relations scale. Specifically, PCA extracted two correlated factors from this scale in the present work (i.e., Security and Tolerance, Sociability and Cordiality). In comparison, earlier research has retrieved three factors from this scale (i.e., Security and Tolerance, Sociability and Cordiality, and Discretion and Civility). Even though PCA and CFA resulted in the deletion of 37 questionnaire items, the measures kept 63 of the original 100 items.

In the present shortened version of the scales, the internal consistency of PREQ and NA was satisfactory, which substantially confirmed previous investigations (10), taking into account that the abbreviated version of the indicators of PREQ and NA included just three or four items, and alpha coefficients of Cronbach's alpha rely on the number of items (i.e., with the same inter-correlation among items, the lower the number of items, the lower the alpha value). More specifically, the alpha values of 18 PREQ indicators varied from 0.719 (External Connections) to 0.876 (Green Areas), with just five being < 0.8 (see Table 4). Furthermore, the NA scale produced a single indicator with strong internal consistency (Alpha = 0.835), demonstrating the scale's unidimensionality.

The connections between PREQ and NA in this investigation confirmed partially what was observed in previous studies conducted in the Italian [see (9)] and Chinese environments [notably in metropolitan Chongqing, see (10)]. For example, the perception of the existence (or lack) of stimulating aspects in the area [labeled negatively as Lack of Opportunities by Bonaiuto et al. (9)] reveals a strong relationship with NA. Another contextual PREQ, the presence (or lack) of calming qualities (i.e., Relaxing vs. Distressing) in the area [classified as Quiet in Bonaiuto et al. (9)], has a strong relationship with NA. As for the Architectural and Urban Planning aspect of PREQ, there is proof of the importance of Building Aesthetics in developing NA. One sociorelational dimension of PREQ, namely Sociability and Cordiality, also confirms a significant association with NA [labeled as Presence of Social Relationship in Bonaiuto et al. (9)].

Taken together, both in the Italian and Chinese urban contexts, the adequacy of Pace of Life patterns (as measured by the Stimulating versus Boring and Relaxing versus Distressing indicators), sociorelational opportunities, and natural and built environment pleasantness appear to be important in determining neighborhood attachment feelings (28, 48). Furthermore, the current study's findings on the association between environmental quality and place attachment corroborated those of earlier research conducted in other cultural settings. For example, place attachment is more excellent for areas of good environmental quality in Israel and England [e.g., (49, 50)]. While Sam et al. (34) found that perceived residential quality was crucial in establishing affective bonds to one's neighborhood (though contextual, functional and human features of neighborhood evaluation are more important than the spatial features) in areas of Turkey. More recently, we found that Chinese residents who perceived good physical environment quality of the residential community would indicate a stronger community identity under the quarantine of COVID-19 (28).

It's worth mentioning that the current research had specific unique characteristics. The perceived quality of *Environmental Health*, in particular, had the most significant association with NA. In other words, in the current COVID-19 challenge (28, 48), individual health-related difficulties seem to be significantly tied to their emotional connection to their living environment. As a result, persons who are more satisfied with the quality of the environment (particularly the perceived quality of Environmental Health) are more likely to form emotional attachments with their home area, as their fundamental security requirements, which are challenged by the COVID-19 pandemic, are met. Overall, these results may assist to explain why each environmental quality aspect is prioritized in encouraging a favorable outcome from the inhabitant-place transaction (27, 32). The relationship between perceived environmental quality and neighborhood attachment investigated in the present work could offer important insights for academics, practitioners, policymakers, as well as urban designers and managers in understanding urban environmental qualities that are most important in influencing place attachment, which could contribute to environmental design or management.

### Implications

In conclusion, a considerable reduction in the number of items for each PREQ and NA scale with reference to previous versions of indicators was a further important result of this study, as it could reduce respondents' fatigue and annoyance with a large number of items. The findings add to the residential satisfaction theory and urge additional environmental psychology researchers to employ simple tools such as the PREQ instrument to assess the quality of the living environment among Chinese people. Our results may aid policymakers in better understanding residents' expectations and requirements, therefore encouraging place identity initiatives in terms of resident satisfaction and contributing to Chinese urban planning and architectural design (28, 48). The findings would support policies targeted at intervening in local environmental aspects in order to increase inhabitants' connection to their community. For example, both the current research and a prior study conducted in China found that the environmental quality of green spaces was positively connected to neighborhood attachment (10, 28). According to recent research (51), streetscape greenery is important in predicting older adults' walking propensity within a certain range. And green areas, together with organization of accessibility and roads, architectural and town-planning space, all three dimensions of architectural and urban planning aspects are important predictors of community residents' identity (28). Therefore, Green spaces, in this respect, provide vital insights for urban designers and politicians to evaluate and create an urban setting with natural green because of the benefits of restoration and health (15, 38). Furthermore, most studies such as the present one were carried out in the western countries, despite the geographical, cultural, and linguistic differences between the Chinese and European contexts, validation work in the Chinese urban context could provide support for the cross-cultural generalizability of the factorial structure of PREQ indicators, which could serve as a foundation for expanding such an approach to other contexts.

### Limitations and future research direction

There are a few limitations in this research that need to be addressed. To begin with, participants were Chinese urban residents who completed our online survey via the disseminated survey link, making it difficult to control those who completed or did not complete the questionnaire. And the online survey would lead to sample deviation to some extent. Future research can enhance the representativeness of samples through the combination of the online and offline surveys. Secondly, the study was based on cross-sectional analysis, future studies could adopt a longitudinal design to improve the predictive validity of indicators of environmental qualities. Moreover, the present work just concentrated on the validation of factorial structures of PREQIs and NA scale through the PCA and CFA techniques. Further research might concentrate on completing the validation process by confirming the instruments' concurrent and discriminant validity in the Chinese culture.

The comparison of people's perceived environmental quality of their area of residence with specialists' technical assessments of the same locations, is one study line that will be explored, to determine when these two evaluations coincide or differ (52). This might help us get a better understanding of subjective and objective environmental assessments, which can then be contrasted and supplemented in environmental management plans and initiatives (33). On a separate level, further study is required to compare studies from Eastern (such as China) and Western nations, which will aid in detecting cultural variations in the evaluation of which factors contribute more to both NA and residential satisfaction.

## Conclusion

The present study had a twofold goal: the creation of shortened versions of the PREQ and NA indicators that were nonetheless reliable; and the validation of factorial structures through the PCA and CFA techniques. In conclusion, the results of this study validated an abbreviated version of the instrument comprising PREQ and NA indicators showing acceptable reliability in the Chinese urban contexts. Moreover, the hypothesis of a positive association between PREQ and NA of inhabitants was also confirmed.

The findings are essential for the residential environment theory study, and they call for further validation and use of the abridged instrument's analysis among residents from various cultural backgrounds. According to this study, environmental psychologists should fight for the subjective well-being of contemporary city dwellers via locations and activities supported by family and neighbors who can help them meet their requirements. PREQ and NA researchers may begin to promote this viewpoint by utilizing empirical data to advocate sensible housing development and welcoming neighborhoods, therefore improving inhabitants' health outcomes. To this end, such a validated instrument can, hopefully, provide a manageable, ease-of-use tool for academics, practitioners, policymakers, and so on.

## Data availability statement

The raw data supporting the conclusions of this article will be made available by the authors, without undue reservation.

## Ethics statement

The present study was conducted according to the American Psychological Association's ethical guidelines for researchers.

## Author contributions

YM commenced the idea of this study, designed the study, collected the data, and revised the manuscript. XL collected the data, analyzed the data, and wrote the manuscript draft. SG collected and analyzed the data. MX revised the manuscript. JZ, RH, and ZZ collected the data. All authors contributed to the article and approved the submitted version.

## Funding

For financial assistance, we appreciate the following funding bodies: the National Natural Science Foundation of China (Nos. 71801180 and 71871201), the Applied Psychology Research Center of Sichuan Province (CSXL-22101), and the Scientific Research Funds of Southwest Jiaotong University (XJXG-2022-002).

## Conflict of interest

The authors declare that the research was conducted in the absence of any commercial or financial relationships that could be construed as a potential conflict of interest.

## Publisher's note

All claims expressed in this article are solely those of the authors and do not necessarily represent those of their affiliated organizations, or those of the publisher, the editors and the reviewers. Any product that may be evaluated in this article, or claim that may be made by its manufacturer, is not guaranteed or endorsed by the publisher.
